# Revisiting the orbital complications of acute rhinosinusitis

**DOI:** 10.1016/j.bjorl.2023.101316

**Published:** 2023-08-30

**Authors:** Wilma T. Anselmo-Lima, Mateus R. Soares, Jefferson P. Fonseca, Denny M. Garcia, Antonio A. Velasco e Cruz, Edwin Tamashiro, Fabiana C.P. Valera

**Affiliations:** Universidade de São Paulo (USP), Faculdade de Medicina de Ribeirão Preto (FMRP), Divisão de Otorrinolaringologia do Departamento de Oftalmologia, Otorrinolaringologia e Cirurgia de Cabeça e Pescoço, Ribeirão Preto, SP, Brazil

**Keywords:** Acute rhinosinusitis, Complication, Orbit, Surgery, Classification

## Abstract

**Objective:**

The present study revisited three classification systems of orbital complications of acute rhinosinusitis (ARS) (Chandler, Mortimore & Wormald, and Velasco e Cruz & Anselmo-Lima) and observed which of them presented the best clinical applicability.

**Methods:**

Clinical data and CT scan findings of patients with orbital infection were retrospectively collected. To compare the three classification systems, we revised and graded all CT images accordingly, and divided the patients into four groups: Eyelid cellulitis (EC), orbital cellulitis (OC), subperiosteal abscess (SA), and orbital abscess (OA). The groups were compared regarding the presence of sinus opacification, the need for hospitalization and/or surgical treatment, and the presence of further complications/sequelae.

**Results:**

143 patients were included. The median number of sinuses involved in patients in the OC, SA, and OA groups was 2.0. ARS was rarely associated with signs of EC (present in both Chandler’s and Mortimore & Wormald’s classifications. The hospitalization rate was significantly lower in the EC group compared to the other three groups. Surgery was performed in all cases in the OA group, in 58.1% in the SA group, 19.4% in the OC group, and 12.5% in the EC group (*p*-value < 0.0001). Complications were present at higher rates in the OA group compared to the other three groups.

**Conclusions:**

ARS was rarely associated with Eyelid Cellulitis. The stratification in the other three groups showed to be clinically relevant. Velasco e Cruz & Anselmo-Lima’s classification system proved valid, simple, and effective for categorizing orbital complications of ARS.

**Level of evidence:**

3.

## Highlights

• Orbital complications should be monitored by clinical signs and CT findings.
• ARS is rarely present in patients with eyelid cellulitis.
• OA is frequently associated with other complications.
• In front of an OA, surgery and IV antibiotics should be immediately started.

## Introduction

The orbit is the most common site for complications from acute rhinosinusitis (ARS).^1^ Some anatomical features support the dissemination and progression of sinonasal infection into the orbit, such as: a narrow ostiomeatal complex, a thinner lamina papyracea, the intimate contact between the orbit and the sinuses, and absence of valves at the ophthalmic venous system.^2^, ^3^

The symptoms observed in orbital complications of ARS include periorbital edema and hyperemia, proptosis, pain during eye movement, ophthalmoplegia, papilledema, and visual loss. In all cases, contrast computed tomography (CT) scans or magnetic resonance imaging (MRI) is mandatory to determine the precise diagnosis and treatment strategy.^4^

The first classification of ARS complications was published in 1937 by Hubert,^5^ which included all the possible complications, be they ophthalmologic or intracranial. However, the authors considered eyelid and orbital complications as the same stage of complication. In 1948, Smith and Spencer^6^ published a new classification system, but they persisted with the same imprecision of group 1, considering eyelid or orbital complications as the same group.^7^

In this aspect, a considerable improvement occurred in 1970, when Chandler et al.^8^ introduced their new classification method. Since CT scans and MRIs were unavailable at the time, the authors proposed this classification based on clinical findings.^9^ They divided the orbital complications of ARS into 5 subgroups: I ‒ inflammatory edema; II ‒ orbital cellulitis; III – subperiosteal abscess; IV – orbital abscess; V – cavernous sinus thrombosis. The definition of each subgroup is detailed in Table 1.

**Table 1:** Definition of each subgroup of each Classification used in this study, as described by the respective authors (Chandler et al.,^8^ Mortimore & Wormald,^9^ and Velasco e Cruz et al.^7^

Classification/subgroup	Definition
**Chandler et al.**^8^	
I – Inflammatory edema	Eyelid edema, with or without edema of the orbital contents. Discrete proptosis can be observed. There are no visual deficits and no restriction of eye movement
II – Orbital cellulitis	Diffuse edema of orbital contents and infiltration in intraorbital fat, without abscess formation. Visual impairment can be present;
III – Subperiosteal abscess	Purulent collection between the periorbita and the orbital wall;
IV – Orbital abscess	Purulent collection within the orbital contents. Exophthalmos and chemosis are more prominent. Ophthalmoplegia and usually severe visual loss are present;
V – Cavernous sinus thrombosis	Posterior extension of phlebitis towards the cavernous sinus. The opposite eye might be involved. Prostration and meningism are frequently observed.
**Mortimore & Wormald**^9^	
1 – Pre-septal infections	a) Cellulitis, and b) Abscess;
2 – Post-septal infections (subperiosteal)	a) Cellulitis, and b) Abscess;
3 – Intraconal post-septal infection	a) Cellulitis: I – Diffuse and II – Confined (orbital apex syndrome), and b) Abscess.
**Velasco e Cruz et al.**^7^	
I – Orbital cellulitis	Diffuse inflammation of the orbital fat, with a poor transition between normal and high-density fat;
II – Subperiosteal abscess	Defined purulent collection between the periorbita and at least one orbital wall adjacent to the sinus;
III – Orbital abscess	Collection with heterogeneous density inside the orbital fat, limited or not by a circular aspect.

In 1997, Mortimore & Wormald^9^ proposed the “Groote Shuur Hospital” classification method, which was based on CT scan analysis. They excluded the term “cavernous sinus thrombosis” since it is an intracranial complication. Also, they used the term “pre-septal” to designate the cases exhibiting periocular/eyelid edema, in which the eye movements were regular. They also divided the cases into cellulitis and abscess, as this is important for the prognosis. The detailed definition of each subgroup is also described in Table 1. The authors described localized intraconal infections (IIIb) as “orbital apex syndrome”, but this term is preferable for infections at the orbital apex (including the superior orbital fissure), no matter the etiology.

These two classifications are the most used in the literature.

Considering that the orbital septum is a fascial layer that limits the anterior orbit and separates the eyelid from the orbit, Velasco e Cruz et al.^7^ proposed a new classification system in 2007.^7^ The authors stated that pre-septal cellulitis should not be considered an orbital complication of ARS since the area involved is the eyelid, not the orbit. This new classification method is straightforward and objective and considers three distinct subgroups: I ‒ orbital cellulitis; II ‒ subperiosteal abscess; and III ‒ orbital abscess. The definition of each subgroup is detailed in Table 1.

An ideal system of classification should be simple, practical, and easily applicable by physicians from different fields (such as ENTs, Ophthalmologists, and Radiologists). Also, it should be based on the gold standard method to diagnose ophthalmologic complications in ARS, i.e., clinical history/findings associated with contrast CT scans and/or MRIs.

The aim of the present study was to revisit these three classification methods, identifying which has the best clinical applicability to evaluate orbital complications of ARS.

## Methods

### Patients

This retrospective cohort study was approved by the local IRB under CAAE nº 40009720.2.0000.544.

All patients diagnosed with acute orbital inflammation (under ICD 10 code-H05) and evaluated at the Clinics Hospital – Medical School of Ribeirão Preto – University of São Paulo from January 1, 1995, to December 31, 2020, had their medical records assessed.

The inclusion criterium was the presence of ocular infections in the medical records. Exclusion criteria were associated diseases (such as immunodeficiencies and fungal infections) or a different cause for acute orbital inflammation, such as inflammatory diseases and/or tumor. The patients with incomplete medical records or absence of CT scans in their electronic files at the Hospital were also excluded.

Patients were considered diagnosed with ARS if they presented a compatible history (including symptoms of rhinorrhea, nasal obstruction/congestion, facial pain, and fever). An otorhinolaryngologist confirmed the diagnosis during hospitalization/admission to the Emergency Unit.

### CT scan evaluation

All the contrast CT scan images obtained during admission were reviewed by two of the observers (M.R.S. and F.C.P.V.) who consistently evaluated if the sinuses (maxillary, frontal, ethmoid, or sphenoid) on the same side as the orbital complication were opacified. The observers scored 1 in cases of partial or complete sinus opacification, and 0 for normal findings. Therefore, the maximum total score for each patient was 4.

**Figure 1:**
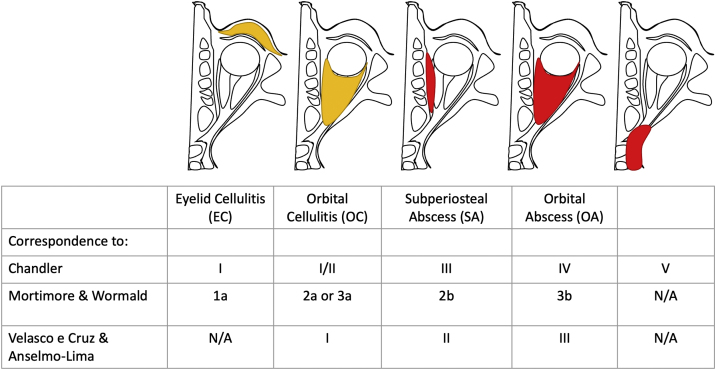
Descriptive illustration, showing the possible ophthalmologic changes described in this study and their corresponding classifications. Eyelid Cellulitis (EC) group (corresponding to Chandler I, Mortimore & Wormald 1, no correspondence to Velasco e Cruz & Anselmo-Lima); Orbital Cellulitis (OC) group (corresponding to Chandler I or II, Mortimore & Wormald IIa or IIIa, and Velasco e Cruz & Anselmo-Lima I); Subperiosteal Abscess (SA) group (corresponding to Chandler III, Mortimore & Wormald IIb, and Velasco e Cruz & Anselmo-Lima II), and Orbital Abscess (OA) group (corresponding to Chandler IV, Mortimore & Wormald IIIb, and Velasco e Cruz & Anselmo-Lima III).

All images were also graded according to all three classification methods evaluated in the present study (Chandler, Mortimore & Wormald, and Velasco e Cruz & Anselmo-Lima). To enable the comparison, we graded the CT scans as follows (Fig. 1) into 4 groups:

• Eyelid cellulitis (EC) group: presence of eyelid edema, with no orbital involvement (corresponding to Chandler I, Mortimore & Wormald 1a, and no correspondence with Velasco e Cruz & Anselmo-Lima)
• Orbital cellulitis (OC) group: increased orbital fat density without organized suppurative collection (corresponding to Chandler I or II, Mortimore & Wormald 2a or 3a, and Velasco e Cruz & Anselmo-Lima I);
• Subperiosteal abscess (SA) group: presence of subperiosteal (periorbital) lump, with an organized purulent collection between the periorbita and the underlying sinus bone (corresponding to Chandler III, Mortimore & Wormald 2b, and Velasco e Cruz & Anselmo-Lima II);
• Orbital abscess (OA) group: a collection with heterogeneous density observed in the orbit, with or without organization (corresponding to Chandler IV, Mortimore & Wormald 3b, and Velasco e Cruz & Anselmo-Lima III).

**Figure 2:**
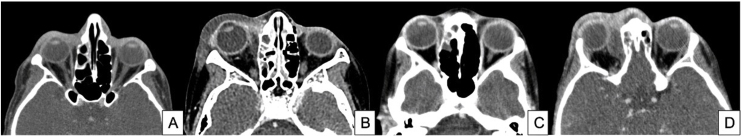
Representative CT scans of the ophthalmologic changes. (A) Eyelid Cellulitis (EC); (B) Orbital Cellulitis (OC); (C) Subperiosteal Abscess (SA); and (D) Orbital Abscess (OA).

The ophthalmologic complications and their corresponding classifications are described in Fig. 1 and can be represented in Fig. 2.

To analyze each classification and to consider it a good one, we addressed two points: (i) all the orbital complications should be related to acute rhinosinusitis; and (ii) each sub-classification should be clinically different from the other (for example, need for hospitalization or surgery).

These four groups were compared one to the other regarding the number of involved sinuses in the CT scans, and the clinical data. The data obtained from the medical records included the patient’s age at the time of hospital admission and sex; presence or absence of sinonasal symptoms consistent with ARS (rhinorrhea, nasal obstruction/congestion, facial pain, and fever); signs of ARS on the CT scans (partial or complete sinus opacification, air-fluid level, and air bubbles within fluid levels in the paranasal sinuses); if the patient required hospitalization for medical or surgical treatment; if the patient underwent surgery, and additional complications and/or sequelae, if present.

The surgery was indicated when the patient presented visual impairment, signs of orbital abscess, or when they showed no improvement within 48 h of intravenous antibiotics.

All data obtained from the medical records and CT scans were inserted in a single digital file.

### Statistical analysis

The categorical data are shown as frequency and percentage. Continuous parametric data are presented as mean and standard error (SE), while non-parametric data are shown as median and interquartile range (IQR). To compare the groups, we used Kruskall–Wallis test, and the comparison of each pair of groups was done using Dunn’s post-hoc test.

The Chi-Squared test was used to compare the four analyzed groups (EC, OC, SA, and OA) regarding sinus opacification, the need for hospitalization and/or surgical treatment, and the presence of sequelae/complications.

Binary logistic regression was used to investigate the need for surgery according to age, sex, and the involved sinus. An ROC curve was also plotted, and the area under the curve (AUC) was determined.

Also, the effect size of the statistical tests was described using Cramer’s V ($\varphi_{C}$) as Small (0.1–0.2), Moderate (0.2–0.3), and Large (>0.3), and Eta squared based on the H-statistic ($\eta_{H}^{2}$) as Small (0.01–<0.06), Moderate (0.06–<0.14), and Large (≥0.14). All the above procedures were conducted using the R software, version 4.1.1 (R Core Team, Vienna, Austria). Significance was set at 5%.

## Results

Based on the inclusion and exclusion criteria mentioned previously, 143 patients were assessed. They were divided into 4 groups, as mentioned in “Methods” section, to observe their correspondence with the three classification systems (Fig. 1).

Regarding demographic characteristics, the eyelid cellulitis (EC) group comprised 56 patients, 30 (53.2%) of which were men, with a median age of 17 ± 37 years. The orbital cellulitis (OC) group consisted of 31 patients, 18 (58.5%) men, with a median age of 9 ± 20.5 years. The subperiosteal abscess (SA) group included 31 patients, 20 (64.5%) men, with a median age of 8 ± 13.2 years. Finally, the orbital abscess (AO) group was represented by 25 patients, 18 (72%) men, with a median age of 13.5 ± 14 years. There was no significant difference among groups regarding sex (*p*-value = 0.43, Cramer’s V = 0.14) and age (*p*-value = 0.16, $\eta_{H}^{2}$ = 0.02).

The sinuses most frequently involved in all groups were the ethmoid and maxillary sinuses, followed by the frontal and sphenoid sinuses (Table 2).

**Table 2:** Frequency of opacification in each sinus. Comparison among groups using the Chi-Squared test.

Opacified Sinus	OA, N(%)	SA, N (%)	OC, N (%)	EC, N (%)	*p-*Value	Cramer’s V
Ethmoid	24 (96.0)	29 (93.5)	29 (93.5)	10 (17.9)	<0.0001	0.78
Maxillary	20 (80.0)	26 (83.9)	28 (90.3)	9 (16.1)	<0.0001	0.689
Frontal	13 (52.0)	17 (54.8)	11 (35.5)	6 (10.7)	<0.0001	0.41
Sphenoid	7 (28.0)	6 (19.4)	4 (12.9)	3 (5.4)	0.0398	0.245

OA, orbital abscess; SA, subperiosteal abscess; OC, orbital cellulitis; EC, eyelid cellulitis.

Bold means - significant *p* (*p* < 0.05).

The median number of partially or totally opacified sinuses was 2.0 in patients diagnosed with OA, SA, and OC. In contrast, most of the patients with EC did not present any sinus opacification; the sinus opacification in this group differed significantly from the other three (*p*-value < 0.0001, $\eta_{H}^{2}$ = 0.46) (Table 3). Direct comparison of each pair of groups revealed a significant difference in the number of sinus opacifications when EC was compared with the other groups (*p*-value < 0.0001 between EC and OA, SA, or OC) (Table 4).

**Table 3:** Descriptive data of sinus opacification in the CT scans (number of affected sinuses) in each group.

Group	N	Mean	SE	Median	IQR
OA	25	2.56	0.18	2.0	1.0
SA	31	2.52	0.15	2.0	1.0
OC	31	2.32	0.14	2.0	1.0
EC	56	0.50	0.15	0.0	0.0

OA, orbital abscess; SA, subperiosteal abscess; OC, orbital cellulitis; EC, eyelid cellulitis. SE, standard error; IQR, interquartile range.

**Table 4:** Comparison among groups regarding the number of sinuses presenting opacification.

Group1	Group2	n1	n2	Statistical difference	*p-*Value^a^	Adj.^b^
OA	SA	25	31	−0.067	0.95	1.00
OA	OC	25	31	−0.584	0.56	1.00
**OA**	**EC**	25	56	−6.062	**<0.0001**	**<0.0001**
SA	OC	31	31	−0.548	0.58	1.00
**SA**	**EC**	31	56	−6.433	**<0.0001**	**< 0.0001**
**OC**	**EC**	31	56	−5.812	**<0.0001**	**<0.0001**

OA, orbital abscess; SA, subperiosteal abscess; OC, orbital cellulitis; EC, eyelid cellulitis. Adj.p, adjusted p.

a Kruskal-Wallis test demonstrated a statistical difference among groups (*p* < 0.0001, $\eta_{H}^{2}$ = 0.46).

b Dunn’s post-test showed that the observed difference was only between EC and the other groups.

Hospitalization occurred either due to the indication of surgery or intravenous antibiotics. Almost all patients with OC (n = 31; 100%), SA (n = 30; 96.8%), and OA (n = 25; 100%) required hospitalization, while this occurred in only 32.1% (n = 18) of the EC group, a significantly lower rate when compared to the other three groups (*p*-value < 0.0001; Cramer’s V = 0.73). Once again, no difference was observed among groups OC, SA, and OA.

The percentage of patients that underwent surgery was 100% in the OA group (n = 25), 58.1% in the SA group (n = 18), 19.4% in the OC group (n = 6), and 12.5% in the EC group (n = 7) (*p*-value < 0.0001; Cramer’s V = 0.68). Dunn’s post-test showed a statistical difference between OA and all the other groups (*p*-value = 0.0022 when compared to SA and *p* < 0.0001 when compared to OC and EC). The indication of surgery was also significantly different in the SA group when compared to patients from the OC (*p*-value = 0.0083) and EC (*p*-value < 0.0001) groups; patients from groups OC and EC did not differ significantly (*p*-value = 0.5858). The patients in the EC group underwent surgery either due to an eyelid abscess or associated dacryocystitis.

The median age was similar between patients that underwent surgery (13 ± 28) and those who did not (11 ± 28) (*p*-value = 0.22).

A total of 17 patients presented sequelae/additional complications in all groups, with 9 (36%) patients in the OA group, 3 (9.7%) in the SA group, 3 (9.7%) in the OC group, and 2 (3.6%) in the EC group. The complications observed in the OA group were cavernous sinus thrombosis, epidural empyema, frontal abscess, and visual loss. The complications present in the SA group were sagittal sinus thrombosis, revisional surgery, and eyelid contraction. In the OC group, one patient required revisional surgery due to recurrence, and another presented retro-orbital abscess and stroke. In the EC group, both patients presented epidural empyema associated with frontal sinusitis.

Binary logistic regression efficiently predicted which factors could be related to a higher chance of requiring surgery, with an AUC of 76.5. This analysis showed that patients with ethmoidal opacification had an OR of 8.5 times higher chances of requiring surgical intervention when compared to opacification in other sinuses (95% CI 1.9–37.5; *p*-value < 0.005). Also, age was associated with surgical intervention, which was more likely indicated in older patients (OR = 1.023 for each year of age; 95% CI 1.001–1.05; *p*-value < 0.05).

## Discussion

Orbital complications of ARS are related to high morbidity, with potential mortality. Therefore, the early and correct diagnosis of these complications is paramount for better managing these patients. Clinical examination associated with contrast CT/MRI of the orbit and sinuses is the gold standard method in cases of suspicion of an orbital complication due to ARS,^1^, ^2^, ^3^, ^4^ as it quickly identifies the complication and differentiates abscesses from cellulitis.

CT scanning is available in most healthcare centers, and it easily identifies and stratifies the complication associated with ARS. Therefore, this exam should be included as an important parameter for a classification for orbital complications of ARS.

Our cohort differs from other published series^3^, ^10^, ^11^, ^12^ since we evaluated all the patients with ophthalmologic complications, regardless of their age. Even so, we observed that this group of complications is most typical among children and teenagers.

Sinus opacification in CT scans and ARS symptoms are rarely observed in patients with eyelid cellulitis. Therefore, Velasco e Cruz et al.’s^7^ classification system is more assertive in selecting only the orbital complications associated with acute rhinosinusitis. In contrast, Chandler’s^8^ and Mortimore & Wormald’s^9^ classification methods describe complications that are not located in the orbital region (the eyelid in both classifications and, in Chandler’s case, the cavernous sinus). Eyelid cellulitis is rarely a complication of acute rhinosinusitis.

Binary logistic regression confirmed the close relationship between ethmoid sinus opacification and the need for surgery, increasing the chance of undergoing surgery by 8.5-fold. This finding has already been described in the literature.^1^ The close relationship and the contact between these sinuses and the orbit are the reason for such association. Also, the thin lamina papyracea increases the rate of this complication at younger ages.^2^

Another important finding in our cohort was the positive correlation between surgical intervention rates and age (OR of 1.023 per year of age). As demonstrated by Zhao et al.,^13^ younger children tend to present a better response to clinical treatment. They are less likely to present with more aggressive complications, with a higher proportion of subperiosteal abscesses. In cases of small and medially located subperiosteal abscesses, a conservative approach is valid.^1^

When considering only the subgroups described in Velasco e Cruz & Anselmo-Lima’s classification, the three did not differ regarding the percentage of patients that required hospitalization, either due to surgical intervention or intravenous antibiotics. However, they did differ significantly in the rate of patients that underwent surgery (100% in the OA group, 58.1% in the SA group, and 19.4% in the OC group) and the percentage of patients with additional complications/sequelae (36% in the OA group, 9.7% in the SA group, and 9.7% in the OC group). These two parameters justify the stratification among these three subgroups and reinforce the clinical relevance of separating the presentations of these three orbital complications.^14^, ^15^

In contrast, eyelid cellulitis was poorly related to sinus opacification in the CT scans, and the patients required lower rates of hospitalization, and lower rates of surgical intervention. All this information infers that eyelid cellulitis has a very different presentation and is poorly associated with ARS symptoms and signs. Also, it should not be considered as “an orbital complication”, for anatomical reasons. This is why it was not included in Velasco e Cruz & Anselmo-Lima’s classification system.

## Conclusion

Symptoms and signs of ARS are rarely observed in patients with eyelid cellulitis.

Velasco e Cruz & Anselmo-Lima’s classification system is reliable, simple, and efficient in stratifying orbital complications based on CT scan findings. This classification also adequately predicted the need for hospitalization, the surgical indication, and the probability of further complications/sequelae in this cohort.

## Funding

The present study was supported by the Coordenação de Aperfeiçoamento de Pessoal de Nível Superior ‒ Brazil (10.13039/501100002322CAPES) ‒ Finance Code 001 – Grant to M.R.S.

## Conflicts of interest

The leading authors of this article are those who described one of the Classifications mentioned in this article, and thus we may consider this as a non-financial conflict of interest.
